# Supplementary material for: Molecular Dynamics Study of Cellulose Nanofiber Alignment under an Electric Field
Source: Polymers (Basel). 2022 May 9;14(9):1925. doi: 10.3390/polym14091925 (PMC9101420; doi:10.3390/polym14091925)
Supplement: Supplementary file 1 [file polymers-14-01925-s001.zip › polymers-1709310-supplementary.pdf]

SUPPORTING INFORMATION

Table S1. Snapshots of CNF alignment along the applied external electric field (z-axis) for the replica MD (0.06 mV/nm_1 and 0.06 mV/nm_2).

Electric field (mV/nm)	0	1 ns	2 ns	3 ns	4 ns	5 ns	6 ns	7 ns	8 ns	9 ns	10 ns	>10ns to 28ns
**0.06_1**	Stable 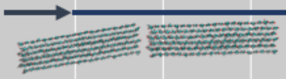				Aligned & stable			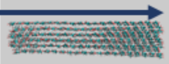 Completely aligned at 1.16ns, slight twisting at 4 ns, lost alignment at 8 ns				-
**0.06_2**	Stable 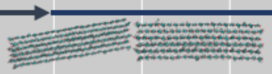				Aligned & stable			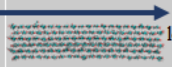 Completely aligned at 1.20 ns, no twisting, lost alignment at 8.01 ns				No realignment after loss of alignment Characterized by mild rotations

Table S2. Electric field along the z-axis: the root-mean-square displacement (RMSD), radius of gyration (Rg), and end-to-end length, averaged over 10 ns of simulation time.

Model	Electric Field Strength (mV/nm)	RMSD average (nm)	Rg average (nm)	End-to-end length (nm)
**Crystalline Cellulose 1β**	No field	0.101 ± 0.02	3.178	11.000 ± 0.01
	0.001	0.105 ± 0.03	3.178	11.002 ± 0.01
	0.005	0.105 ± 0.03	3.178	11.006 ± 0.02
	0.01	0.105 ± 0.02	3.178	11.003 ± 0.02
	0.06	0.105 ± 0.03	3.178	11.005 ± 0.02
	0.09	0.106 ± 0.02	3.178	11.006 ± 0.02
	0.2	0.111 ± 0.02	3.178	11.008 ± 0.01

*Zero standard deviation for Rg

Table S3. Averages of the reproducible hydrogen bonds, radius of gyration, and end-to-end length at the 0.06 mV/nm electric field with repeat productions (0.06 mV/nm_1: the first MD production 0.06 mV/nm_2: the second MD production).

Model	Electric Field Strength (mV/nm)	Hydrogen Bonds Overall	Average Rg (nm)	End-to-end length (nm)
**Crystalline Cellulose 1β**	0.06mV/nm_1	766 ± 12	3.178	11.005 ± 0.02
	0.06mV/nm_2	765 ± 14	3.178	11.006 ± 0.04

Table S4. Electric field along the x-axis: RMSD, the radius of gyration (Rg), end-to-end length, and hydrogen bond population.

Model	Electric Field Strength (mV/nm)	RMSD average (nm)	Rg average (nm)	End-to-end length (nm)	Hydrogen bond population (%)
**Crystalline Cellulose 1β**	0	0.104 ± 0.02	3.178	11.000 ± 0.01	758.538 ± 12.83
	1	0.112 ± 0.02	3.178	11.006 ± 0.01	752.930 ± 13.86
	2	0.123 ± 0.02	3.1782	11.009 ± 0.02	749.402 ± 15.49
	4.5	0.128 ± 0.03	3.178	11.008 ± 0.01	748.882 ± 13.44

*Zero standard deviation for Rg

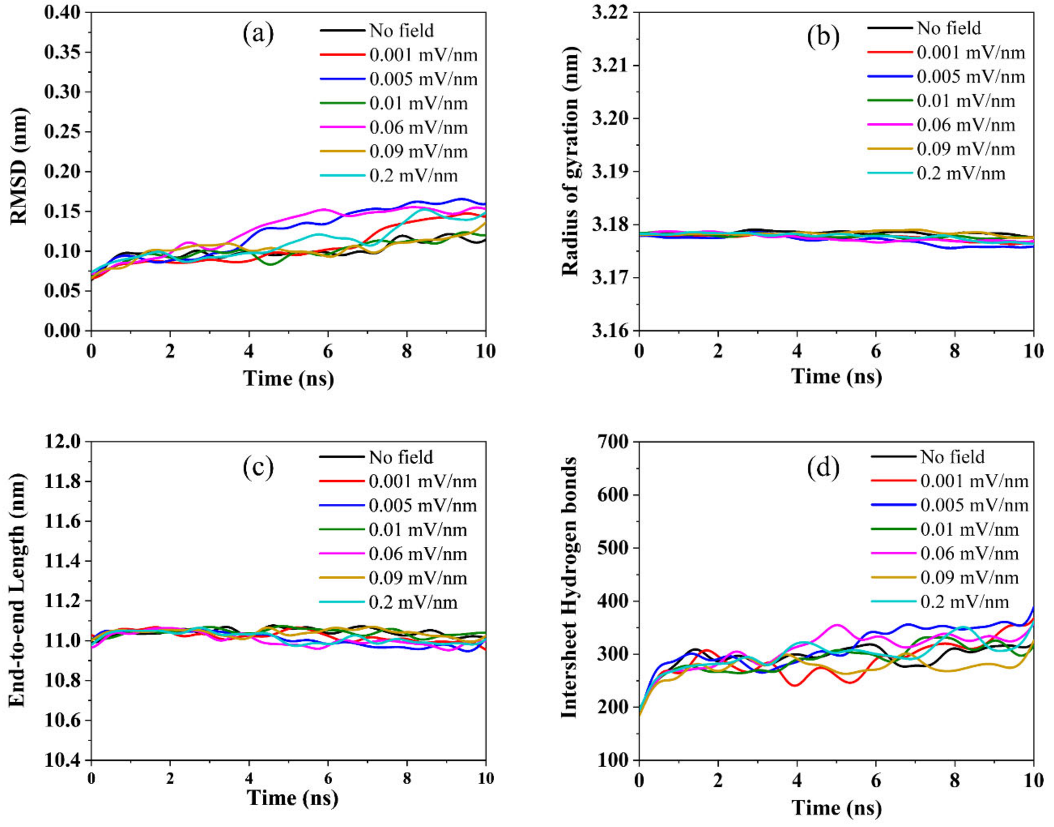

Figure S1. Electric field along the z-axis: Time evolution of (a) the root-mean-square deviation (RMSD), (b) radius of gyration, (c) end-to-end length, and (d) intersheet hydrogen bonds.

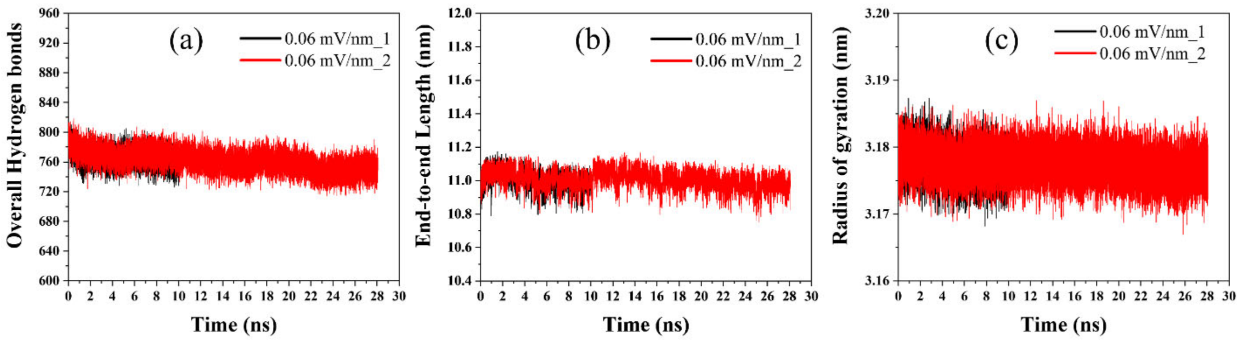

Figure S2. Reproducible hydrogen bonds, the radius of gyration, and end-to-end length for the replica MD (0.06 mV/nm_1 and 0.06 mV/nm_2).

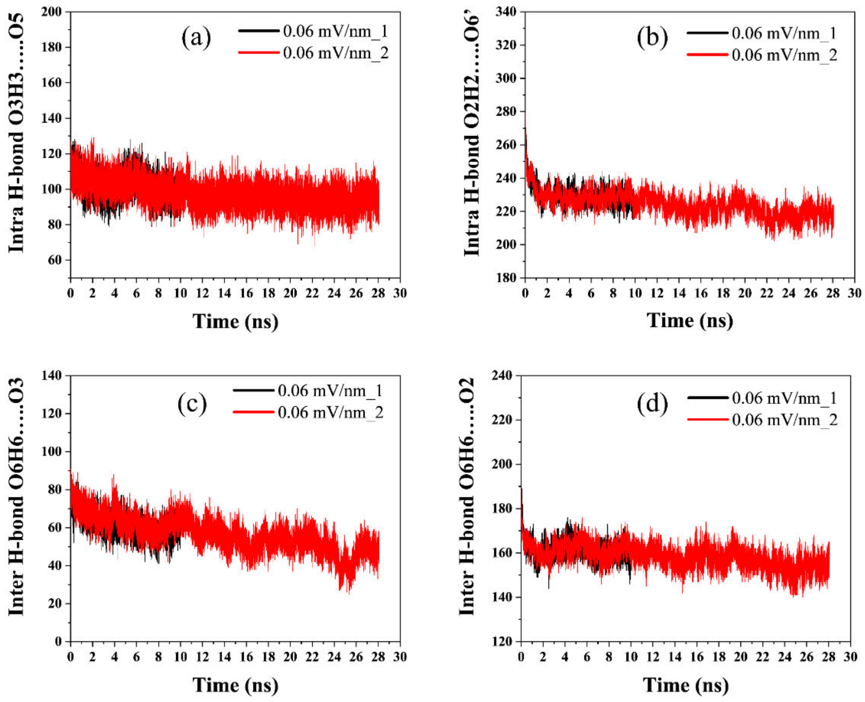

Figure S3. Reproducible hydrogen bonds for the replica MD (0.06 mV/nm_1 and 0.06 mV/nm_2): intra H-bond O3H3…..O5 (a), intra H-bond O2H2…..O6′ (b), inter H-bond O6H6…..O3 (c), and inter H-bond O6H6…..O2 (d).

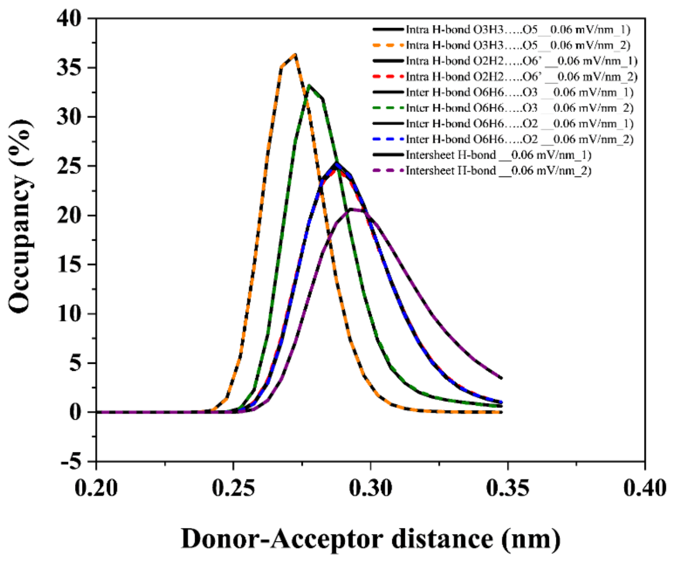

Figure S4. The hydrogen bond formation distance between the donor and acceptor atoms of cellulose and their occupancy under the external electric field for the replica MD (0.06 mV/nm_1 and 0.06 mV/nm_2).

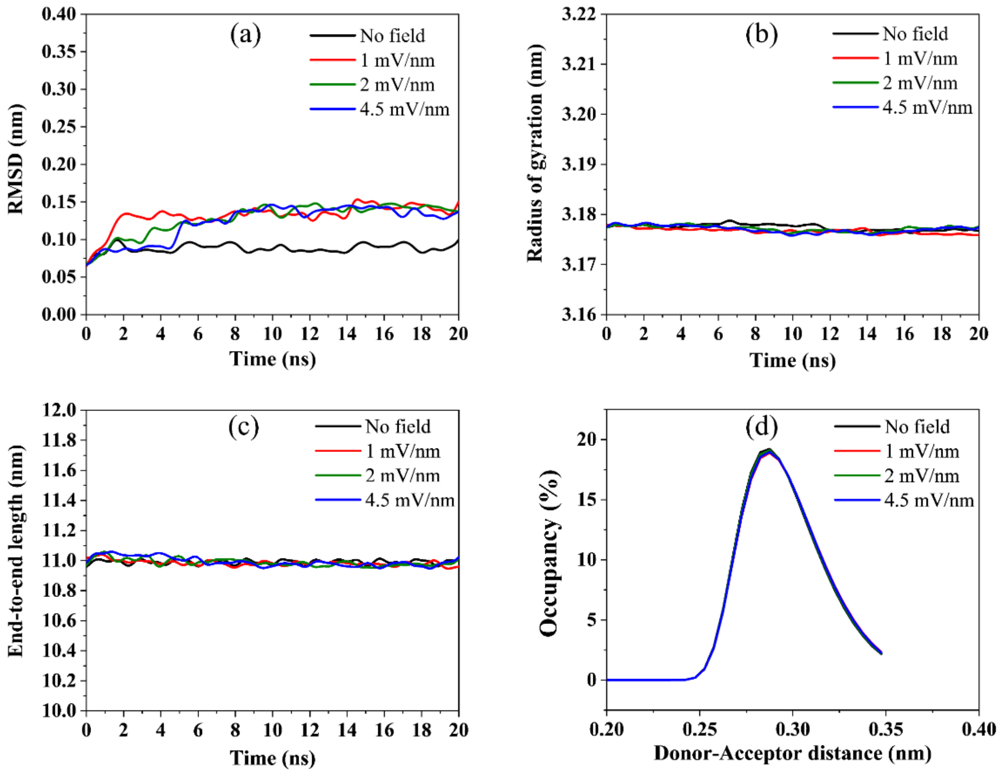

Figure S5. The electric field along the x-axis (a) RMSD, (b) radius of gyration, (c) end-to-end length, and (d) donor acceptor distance and occupancy of hydrogen bonds.
